# Numerical Methods in Studies of Liquid Crystal Elastomers

**DOI:** 10.3390/polym13101650

**Published:** 2021-05-19

**Authors:** Madjid Soltani, Kaamran Raahemifar, Arman Nokhosteen, Farshad Moradi Kashkooli, Elham L. Zoudani

**Affiliations:** 1 Department of Mechanical Engineering, K.N. Toosi University of Technology, Tehran 19991-43344, Iran; farshad.moradi@email.kntu.ac.ir (F.M.K.); elham.lzn@yahoo.com (E.L.Z.); 2 Department of Electrical and Computer Engineering, University of Waterloo, Waterloo, ON N2L 3G1, Canada; 3 Centre for Biotechnology and Bioengineering (CBB), University of Waterloo, Waterloo, ON N2L 3G1, Canada; 4 School of Optometry and Vision Science, Faculty of Science, University of Waterloo, 200 University Ave. W, Waterloo, ON N2L 3G1, Canada; kraahemi@gmail.com; 5 Advanced Bioengineering Initiative Center, Computational Medicine Center, K.N. Toosi University of Technology, Tehran 19991-43344, Iran; 6 College of Information Sciences and Technology (IST), Data Science and Artificial Intelligence Program, Penn State University, State College, Pennsylvania, PA 16801, USA; 7 Department of Chemical Engineering, Faculty of Engineering, University of Waterloo, 200 University Ave. W, Waterloo, ON N2L 3G1, Canada; 8 Department of Civil and Mechanical Engineering, University of Missouri-Kansas City, Kansas City, MO 64110, USA; armannokhosteen@gmail.com

**Keywords:** liquid crystal elastomer, numerical methods, finite element method, Monte Carlo method, molecular dynamics method

## Abstract

Liquid crystal elastomers (LCEs) are a type of material with specific features of polymers and of liquid crystals. They exhibit interesting behaviors, i.e., they are able to change their physical properties when met with external stimuli, including heat, light, electric, and magnetic fields. This behavior makes LCEs a suitable candidate for a variety of applications, including, but not limited to, artificial muscles, optical devices, microscopy and imaging systems, biosensor devices, and optimization of solar energy collectors. Due to the wide range of applicability, numerical models are needed not only to further our understanding of the underlining mechanics governing LCE behavior, but also to enable the predictive modeling of their behavior under different circumstances for different applications. Given that several mainstream methods are used for LCE modeling, viz. finite element method, Monte Carlo and molecular dynamics, and the growing interest and reliance on computer modeling for predicting the opto-mechanical behavior of complex structures in real world applications, there is a need to gain a better understanding regarding their strengths and weaknesses so that the best method can be utilized for the specific application at hand. Therefore, this investigation aims to not only to present a multitude of examples on numerical studies conducted on LCEs, but also attempts at offering a concise categorization of different methods based on the desired application to act as a guide for current and future research in this field.

## 1. Introduction

LCEs are a type of synthetic materials with specific features that make them appropriate for use in many applications [1,2,3,4,5]. LCEs are made of polymers that have been cross-linked with liquid crystalline molecules (known as mesogens). Thus, they possess both the elasticity of polymers and the properties of liquid crystalline materials [6]. The polymer chains can rearrange into anisotropic and random-coiled arrangements in the anisotropic and isotropic states, respectively, based on the mesogenic orientation. The features of LCEs are critically dependent on their preparation mechanism [7]. The synthesis procedure of the LCE samples encompasses two polymerization stages. The first step is polymer strands generation and the next one is cross-linking of them [7]. A monodomain sample with aligned mesogenic orientation can be generated by applying an appropriate external field through the second stage of the polymerization, while without such precautions, polydomain LCE with opaque appearance is created [7]. By applying large enough uniaxial stress, LCE materials will go through the polydomain–monodomain transition [7,8]. According to the structure of LCEs, as they consist of a polymeric network with mesogenic units with a strong coupling between these two, any change in mesogenic orientational order can result in a considerable change in the network of the elastomer, and in turn any stress in the polymeric network has an effect on orientational order of the liquid crystal [9]. For instance, in a nematic LCE, isotropic phase transition produces dramatic strain changes to the nematic LCE, thus this phenomenon makes it a good candidate for artificial muscles and soft actuators applications, conversely applying strain to the nematic elastomer, induces changes in the magnitude of the orientational order, providing the interesting phenomenon of semi-soft elasticity [10].

A comprehensive review on the physical properties and molecular theories governing the phase transitions and molecular behavior of LCEs can be found in [11]. The ability to switch between anisotropic and isotropic states, once influenced by stimuli such as light, heat sources, electric and magnetic fields, has made LCEs the target of many research studies with the goal of not only understanding their mechanical behavior, but also developing applications and the modeling tools needed to predict the mechanical behavior of samples when put to use in different real-world scenarios.

A great number of published researches regarding LCEs is concerned with the experimental study of their behavior and development of their applications. These include studies on the development of artificial muscles [12], next generation solar collector technology [13], and a variety of other applications [14,15,16]. As in any other field of engineering, the ability to model and predict the behavior of materials under various working conditions is an important task. Because of the highly complex and nonlinear behavior exhibited by LCEs, the applicability of analytical methods is limited; therefore, computer simulations are carried out using numerical techniques.

A review of the existing research shows that the most popular and mainstream methods are the finite element method (FEM), Monte Carlo method (MC), and molecular dynamics (MD). It is worth noting that other numerical strategies are also employed such as non-local continuum modeling and thermomechanical models, but are less frequently used than the three aforementioned methods. At the turn of the current century, Zannoni [17] conducted a review on the state of computer modeling methods used for studying LCEs, with a focus on molecular level models. The author’s findings showed that the mainstream methods are comprised of MD and MC. Although procedurally different, the former being concerned with equations of motion and the latter with average properties obtained from equilibrium configurations, both methods evaluate the intermolecular interactions and energy, within a given sample. The author’s results showed that for studying smectic, nematic, and columnar liquid crystals systems, models describing potentials based on the Gay-Berne (GB) molecular level interaction were very useful. It was concluded that by utilizing suitable combinations of various ellipsoidal GB and spherical Lennard-Jones particles, molecular structures with increasing complexity could be simulated, e.g., asymmetric molecules [18,19] or when flexible chains were included [20]. In addition, the basic GB system could also be modified to model quadrupolar [21] or hydrogen bond interactions [22].

This paper presents a comprehensive literature review to provide insight into the modeling methods used to study LCEs. The analysis is meant to serve as a guide for choosing suitable modeling approaches for problems of various levels of complexity. In this paper, the authors attempt to provide an overarching view of the numerical methods used to study LCEs. The primary objectives are as follows: (a) categorize the main numerical strategies used by researchers, (b) provide examples of how they have been utilized, and (c) provide a comprehensive guide for future researchers on the specifics involved with each introduced method. Therefore, this review paper is partitioned into eight sections. Section 1 deals with introductory remarks. LCE modeling approaches with elements required for the numerical modeling of these materials are presented in Section 2. Section 3, reviews research done using FEM. Similarly, Section 4, Section 5, and Section 6 concern MC, MD, and other numerical methods, respectively. In Section 7, a guide will be provided on how to use each of the aforementioned methods, based on how previous researchers have employed them in the past. Finally, in Section 8, the paper closes with conclusions.

## 2. LCE Modeling Approaches

During modeling process, LCE design needs to be converted into computational domain in order to be utilized for running mathematical modeling or numerical simulations. When computational domain is created, all the properties attributed to the LCEs, e.g., sample dimensions, Young’s modulus (E), Poisson’s ratio (ν) or specific heat of the material, stress–strain curve, interaction energy between liquid crystal molecules (in molecular-based simulation studies), etc., need to be chosen following the required conditions in the modeling stage. Accurate simulation tools pave the way of design and engineering of new devices. In fact, these tools provide the possibility of exploring the dependence of the outcome on the design factors and understanding the differences exist between analytical simulation modeling and experimental one [23].

At the stage of the modeling of LCEs, numbers of tools have been proven potentially powerful in modeling and simulation of these materials. FEM strategy has been commonly used for understanding the physics of the LCEs. In other words, by applying the material properties along with set of governing equations (e.g., stress–strain relations, energy equation, etc.) and boundary conditions, macroscopic behavior of LCE, such as deformation, dynamic of shape evolution in nematic order [24], instability [25], and dynamic response when exposed to external stimuli are envisioned [26]. Altogether, this method allows us to understand the fundamental physics that governs the mechanics of nematic elastomers which can be controlled by both nematic order and mechanical strain [27].

It is worthwhile noting that, although the general behavior of LCEs is qualitatively understandable, having information at molecular level is necessary, in particular, for LCE applications as sensors or actuators a deep insight into molecular stage is needed, and this can be achieved through molecular-based simulations [28]. Two approaches, namely lattice and off-lattice models, have been commonly used in studying molecular scale simulation studies. Lattice models mostly deal with orientational properties [28]. Due to the lack of translational degrees of freedom, they cannot easily handle phases involving positional and orientational order [28], as a result, more realistic off-lattice models with translational degrees of freedom seem to be a better choice for specific purposes, e.g., modeling the smectic phases [28].

Owing to the difficulties associated with the equilibrating of 3-D network which result from topological constraints of the bonds, creating a system with a capability of modeling mesogenic nature of the system at molecular stage is rather challenging [28], however, such difficulties can be smoothed by the use of molecular simulation strategies. Computer simulation methods, like MC and MD, have been commonly used for modeling and analysis of the LCE from molecular viewpoint. The MD method mainly concerns with equations of motion while the other one, MC, deals with average properties obtained from equilibrium configurations. By the use of molecular-based simulation techniques and defining the intermolecular interactions (interaction potential energy) between mesogens, phenomenon like polydomain–monodomain transition can be modelled at the molecular level [8]. In that way, several intriguing details related to this mechanism, including the molecular origin of the transition or how strain application may influence the size and orientation of the domain, can be understood [8].

In the following sections, we will elaborate on these computational methods with their applications mirrored in different case studies.

## 3. Finite Element Analysis (FEA)

### 3.1. Commercial Software

Finite Element Analysis (FEA) is a numerical analysis technique used for simulations involving physical phenomena that can be described using partial differential equations (PDEs) [29]. In this method, a given geometry is discretized into small subsections and approximate the PDEs on each subsection (referred to as elements) using numerical model equations i.e., Interpolation functions are used to interpolate the field variables over the element, which can be solved using numerical methods. Therefore, the solutions to the numerical equations will ultimately constitute an approximation of the solution of the original continuous PDEs on the given domain [30,31,32]. FEM proves to be a powerful tool in solving complicated differential equations. Two features of FEM have contributed to its popularity as a widely used numerical tool for a vast array of engineering problems namely: (a) good precision is provided via the piece-wise approximation of physical fields on finite elements and (b) the approximations are local which leads to systems of sparse equation for a discretized problem. This is a great contributor to the ability to handle large number of nodal unknowns [33].

The authors’ review on the existing literature shows that among the said methods, FEM has been used extensively by researchers. FEM has been used to investigate, among other things, the opto- and thermo-mechanical behavior of photochromic liquid crystal elastomer, pattern formation in LCEs, modeling instability and inhomogeneous deformation, and to predict bending patterns and origami behavior of LCEs.

Lin et al. [34] studied the Quasi-soft opto-mechanical behavior of a two dimensional (2D) rectangular beam using commercial FEM software, ABAQUS 6. 12 © Dassault Systèmes, RI, USA, 2012. Instead of using the classical Beer’s equation for light absorption, the authors used an equation proposed by [35] and subsequently derived the linear stress strain relation for soft LCEs [36] and simulated the light induced bending in photochromic LCEs. User defined functions were programmed into the software in order to carry out the simulations and a complete description of the used methodology can be found in the authors’ original manuscript. The authors were able to demonstrate that in the absence of illumination, due to the soft behavior of LCEs, shear stress perpendicular to the director vanishes. However, when the material is laminated, shear stress becomes proportional to the rotation of the director. Another important result stemming from the authors’ findings is that, due to the soft behavior of neo-classical LCEs, Euler–Bernoulli beam theory assumptions should not be directly implemented for modeling light induced bending behavior.

**Figure 1:**
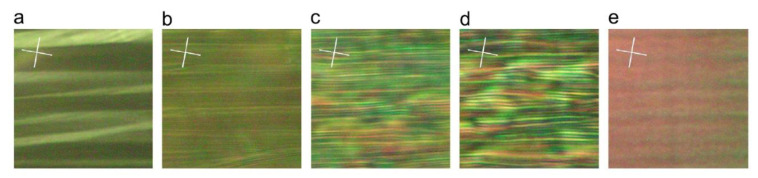
Stripes at the threshold strain of sample, with the final cross-linking done in the isotropic phase, as seen under the crossed polarized microscope. The orientation of crossed polars is indicated and the length of white lines corresponds to 10μm. Initial director orientation was in the vertical direction, and stretching was performed in the horizontal direction. Close to the transition (just below 77.4 °C) a very fine director modulation can be seen. (**a**) T = 70.0 °C, (**b**) T = 75.0 °C, (**c**) T = 77.0 °C, (**d**) T = 77.3 °C, (**e**) T = 77.4 °C [37] (Reprinted with permission from [37]).

De Luca et al. [37] used COMSOL Multiphysics^®^ 4. 3a, COMSOL Inc., Burlington, MA, USA, in conjunction with data obtained from stretching experiments, to study the appearance of sub-stripe patterns in sheets of nematic elastomers. The samples used were consisted of side-chain LCEs with a backbone consisting of a siloxane-based polymer and a 3-but-3-enyl-benzoic acid 4-methoxy-phenylester as the mesogenic moiety. The length of the samples was approximately 5 mm and their width was about 3 mm with thickness of about 200 µm. The samples were placed in a test cell equipped with temperature stabilization, a force gauge and a micrometer for controlling both stress and strain. Figure 1 displays the stretched sample, in the nematic phase, in the direction which is perpendicular to the nematic director. It is observed that at the critical strain λc, stripes are formed. The average size of these shear domains is around 10 μm and they show a random pattern of alternating shear and alternating director orientation.

By using neo-classical rubber elastic theory developed by Warner and Terentjev [38], the authors were able to derive a linear model for carrying out their numerical analysis on nematic elastomers. Using linear models has the advantage of being able to exploit linear superposition of effects. Once linear expressions were derived, they were fed to COMSOL 3.3a model, COMSOL Inc. Burlington, MA, USA, using user-defined subroutines and the numerical simulations were carried out. The results showed that authors’ hypothesis that the sub-stripe behavior, caused by defects and heterogeneities present in nematic elastomer samples, can produce numerical data that are in good agreement with experimental data.

**Figure 2:**
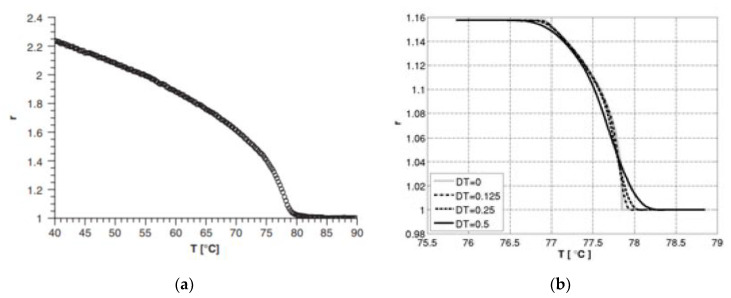
(**a**) [40], shows the correlation between r = ${\left(\frac{L}{L_{ISO}}\right)}^{3}$ and T °C, and how it depends on the nematic order parameter. Note that L denotes the length of a sample when it is in the nematic phase and LISO denotes the sample’s reference length, when it is 12 °C above threshold temperature of 78 °C. (**b**) shows the good agreement between numerical results and experimental data close to the transition temperature [39]. DT accounts for the random fluctuations added to the threshold temperature, which fluctuates specially (Reprinted with permission from [39,40]).

**Figure 3:**
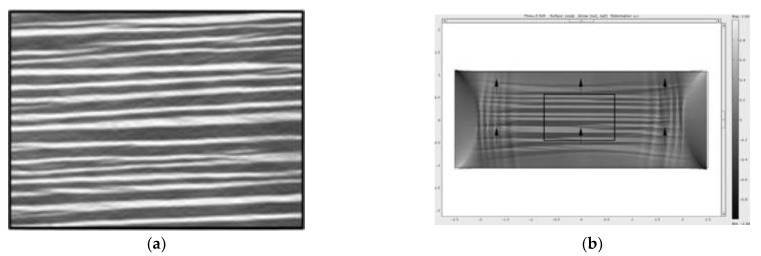
(**a**,**b**) Compare experimental data numerical results, obtained from a stretching experiment, are shown in (**a**,**b**), respectively [39]. The stretching direction was horizontal (Reprinted with permission from [39]).

A linearized form of the neo-classical rubber elastic theory Warner and Terentjev [38] model was also used by De Luca et al. [39] to perform numerical simulations on cooling and stretching experiments, in the direction perpendicular to that of the director at cross-linking. These simulations were carried out in order to study the isotropic to nematic phase transitional behavior of nematic elastomers. Using the linear derived model, COMSOL Multiphysics^®^ 4.3 software, COMSOL Inc. Burlington, MA, USA, was used to study plane-strain deformations. The authors further developed their model by adding a spatially random temperature fluctuation or “numerical noise”, denoted as DT, to the threshold temperature, denoted by T_IN_, to allow for phase transition to happen at different temperature in different points of the sample. The results from numerical modeling for both stretching and cooling experiments were compared with data from experiments [38,40] and good agreement was observed between the two, as can be seen in Figure 2 and Figure 3. Additionally, lateral shear was not observed due to the presence of the clamps, therefore the material develops a micro-structure. The boundary condition of this structure is influenced by the presence of stripes. With this model, unfortunately, complex behavior of LCE cannot be described because of the hypothesis of plane–strain deformation.

A FEM model was built by [41], using ANSYS^®^ software, Canonsburg, PA, USA, to bridge the gap between elastomer physics and device engineering and design. An empirical model was developed by the authors, for describing the material deformation, using experimental actuation data. The basic concept of the model is as follows: the absorption properties of carbon nano-tubes (CNTs) causes the irradiated light on the sample to be absorbed and consequently, turned into heat. Thus, the CNTs act as internal heat generators. Finally, the opto-mechanical behavior, i.e., the mechanical deformation induced by light as stimuli, of an LCE-CNT system was evaluated and the mechanical response of the said system was optimized. The LCE-CNT system structure which was modeled was an LCE-CNT film in the shape of a cantilever structure with 1 mm length, 0.6 mm-wide, and with a thickness of 0.4 mm. Both 2D and 3D geometries were studied. Effective physical properties were considered for the entire specimen under investigation. Results showed that preliminary results obtained from simulations, using the authors’ proposed model, were in agreement with the experimental data [42]. Another advantage of the developed numerical model was that it could provide the researchers with data that was unattainable via experiment such as, the temperature distribution inside the specimens and also predicting the temperature evolution for any given point in the model geometry.

An et al. [25] describe a FEM scheme, using user-defined subroutines in ABAQUS © Dassault Systèmes, Johnston, RI, USA, for accurately modeling the inhomogeneous deformation and instability of a microfluidic valve, which was made of a LCE beam. In addition, a laminated LCE sample, was also studied. The material used for laminating the sample was a nanoscale poly styrene (PS) film. The model was able to predict the onset of instability. Additionally, the wavelength and amplitude evolution of instability, along with the instability patterns, were also modeled. The numerical results were consistent with analytical data and other previously reported experimental data [4].

**Figure 4:**
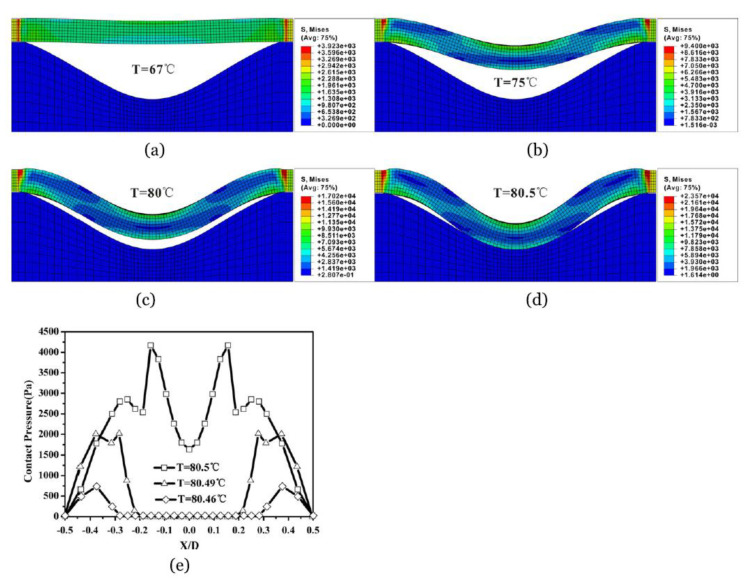
(**a**–**d**), show the deformation, distribution of stress, and contact pressure of the LCE microvalve, at various temperatures. Note that the color intensity represents the intensity of the local stress distribution. (**e**) illustrates both the contact region and contact pressure between the LCE valve and surrounding silicon wall. Note that the temperature is close to phase transition temperature [25]. Here, X is the distance measured from the central point of the contact and D is the length of the contact area of the LCE beam [25] (Reprinted with permission from [25]).

**Figure 5:**
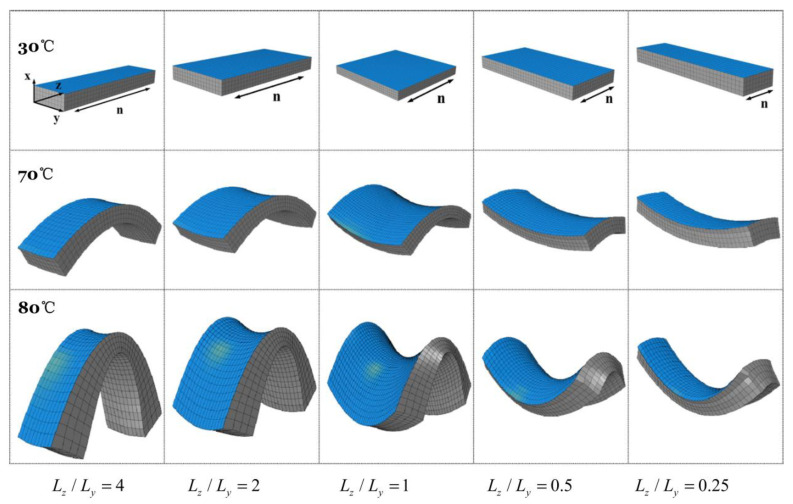
Buckling phase diagram of LCE–PS bilayers with different aspect ratios and temperatures, ranging from 30 °C (reference temperature) 80 °C (Reprinted with permission from [25]).

The data obtained for valve actuation and LCE-PS buckling are shown in Figure 4 and Figure 5, respectively. The authors state that the success and accuracy of the simulation scheme are due to a phenomenological constitutive law adopted in the developed scheme. With regard to most of LCEs reported in the study, which were monodomain and were synthesized using the two-step approach [25], the discrepancies between the numerical results and the experimental data is quite small and this is therefore ignorable. Furthermore, due to the proposed scheme being generic, in order for it to be used for studying other LCEs, not considered in the study, all that is needed is that a proper specific constitutive law, or phenomenological approximation, be provided.

**Figure 6:**
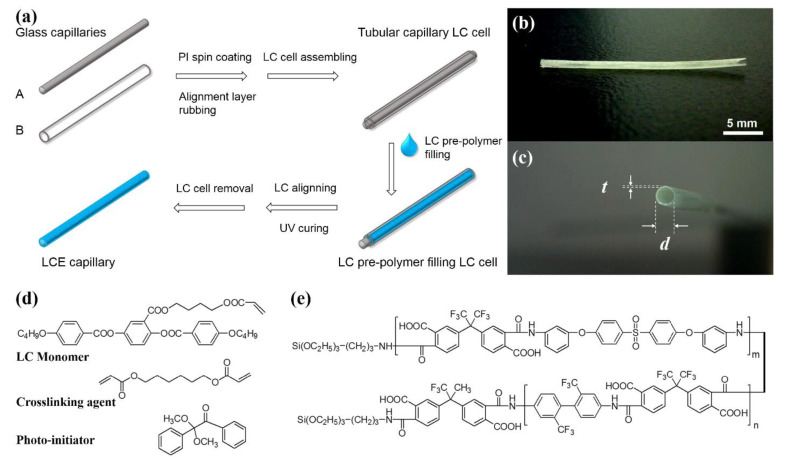
(**a**) Schematic view of LCE capillary fabrication via PI spin-coating, LC cell assembling, alignment layer rubbing, LCE precursor filling and photo-induced polymerization/crosslinking [43]. (**b**,**c**) photographic images of as-prepared LCE capillary with dimensions (Length = 27 mm, Diameter = 0.9 mm and wall thickness = 70 µm); (**d**) chemical structure of the monomer, crosslinking agent and photoinitiator; (**e**)chemical structure of polyamic acidused for preparing polyimide alignment layer (Reprinted with permission from [43]).

For the first time, in a study conducted by Liu et al. [43], a LCE capillary was fabricated, with biomimetic peristaltic functionality, in order to simulate the crawling behavior of earthworms. Therefore, a specially designed liquid crystal (LC) cell was prepared, comprised of two coaxial glass capillaries. The inner surface of the cells was coated with polyimide alignment layers. The methods used in the fabrication of the capillaries are described in detail in the authors’ original manuscript [43]. Figure 6 illustrates the fabricated capillary. In order to gain a further understanding of the peristaltic crawling process, FEA was used for simulating the temperature distribution in the LCE capillary under the influence of the moving heat source.

A shell conducting model, implemented in COMSOL Multiphysics^®^ 5.2a COMSOL Inc., Burlington, MA, USA, was used for carrying out the simulations. This model accounted for the movement of the heat source. Results show a reversible contraction/expansion and relative length changes of 16% was observed at the nematic/isotropic phase transition of the LCE capillary. The practical fabrication and specific actuation mode offer promising prospects for usages including actuators, microrobots, and other devices requiring peristaltic movements. The results showed that as the speed of the heat source increases, so does the peristaltic speed. However, this speed is limited by both the heat exchange and cooling rates, which are integral to the reversible phase transition process. Therefore, the peristaltic crawling speed can be augmented by a more effective heat transfer process.

Keip and Bhattacharya [24] introduced a different approach, based on phase-field model, for the continuum mechanical modeling of nematic LCEs. This model was implemented into FEM, using FEAP (Finite Element Analysis Program)—written by Prof. R. L. Taylor, Berkley, USA—and was used to carry out simulations on the domain evolution of a square LCE sample clamped on one side and free on all other three sides.

Oates et al. [44] developed a phase field predictive modeling framework to model monodomain and poly-domain structural evolutions in LCs as a result of large deformations. In this study, a thermo-mechanical energy function for elastomer network is coupled with a LC free energy function which accounts for mono and poly-domain deformations. The authors reported that their formulation has led to a reduced order model leading to a phase field formulation, coupling the effects of elastomer mechanics with the evolution of polydomain liquid crystal structures, which can reduce computational costs. After the mathematical formulation has been developed, the authors used COMSOL 3.5, COMSOL Inc., Burlington, MA, USA, for numerical implementation on a 2D model. The spontaneous evolution of strain and domain structure, caused by different stimuli, was predicted for a monodomain LCE. In addition, it was shown that, due to Frank elastic energy, in cases of large stretching and temperature changes, domain structures begin to form. Although only qualitative predictions had been considered, the authors showed that that finite deformation coupling was able to provide an insight into soft elasticity. The results showed that the deformation caused by pseudo-directors was similar to the stresses caused by electric field, in dielectric elastomers. However, due to the nonconvex nature of the LC energy function, the coupling process involved between LC and elastomer is more complex and hence is modelled by considering higher order anisotropic effects. Once anisotropy is introduced into the energy function, the pseudo-director orientation determines the deformation. Additionally, because polydomain structures were considered, this approach was able to define an additional set of stress relations.

Another application of commercial FE software is the study of wrinkling behavior in LCEs. Plucinsky et al. [45] studied the wrinkling behavior in sheets of stretched nematic elastomer. In order to do so, the authors took two contributing sources of instability into account namely, (a) material instability, which causes formation of fine-scale microstructure and (b), structural instability which causes fine scale wrinkles. Therefore, a multiscale view was adopted, and a theory was developed. The result is a Koiter-type theory [46,47,48]. It has two terms: (a) 2D or plane stress decrease of the relaxed energy of DeSimone and Dolzmann [49] and (b) bending. The authors studied the response of taut and appreciably stressed sheets of nematic elastomer, both analytically and numerically. The numerical study was carried out using ABAQUS software© Dassault Systèmes, Johnston, RI, USA. The authors have shown that through the formation of microstructures, wrinkling can be suppressed in nematic elastomer sheets, due to the modification of expected stress states. Finally, the results were compared with those of Kundler and Finkelmann [50] and showed that their developed model was able to accurately recreate experimental results.

Open-source software packages have also been used for LCE simulations. One such example is Salomé [51] which was used by [52] to perform FE elastodynamics simulations on dual-phase nematic ribbons. According to the authors, their research was the first instance of FE elastodynamics being used for the study of actuation behavior in dual-phase elastic solids. The employed method was based on the work done by [26] with two additions: (a) defining the order parameter and nematic directors in each mesh element (tetrahedral in this case) used for discretizing the sample geometry, and (b) insuring that enough mesh elements were used so that the directors transitioned smoothly. The results were able to show that the employed method was adequate for modeling the behavior of dual-phase elastic solids from both a design standpoint and for investing their shape morphing behavior under external stimuli.

Recently, in a study conducted by Brighenti et al. [53], a micromechanical-based model, focusing on the evolution of the LCE network chain distribution tensor, has been proposed. Through this study, firstly, a statistical definition of microstructure nature of liquid crystal elastomers was presented then a quantitative, physics-based micromechanical model was used to evaluate the mechanical reaction of LCE-based elements to thermal stimuli. They looked at two cases of FE numerical simulation study, (a) simple case of monodomain nematic elastomer sample and (b) thermal change-induced bending reaction of soft actuators with bilayer hinges. The obtained FE simulation results were validated by both experimental data from [54] and analytical results given by [55]. Results reveal that the bending behavior of LCE bilayer actuators can be effectively modeled using a statistical mechanics technique. Based on the validation results, there was a good agreement between this proposed model and experimental data.

### 3.2. In-House Coding

The use of FEM is not limited to commercial software packages. Many researchers have opted for using their own, in-house developed, codes. DeSimmone [56] presented a coarse-grain model whereby using the definition of energy density, fine-scale oscillating behavior of nematic elastomers could be accurately modeled through FEM analysis. The method was applied to studying stretching behavior of thin elastomeric sheets, held between two clamps, and was shown to resolve the macroscopic and microscopic behavior of the elastomers. The author provides detailed mathematical analysis of how free-energy density formulation can be derived in the original manuscript.

**Figure 7:**
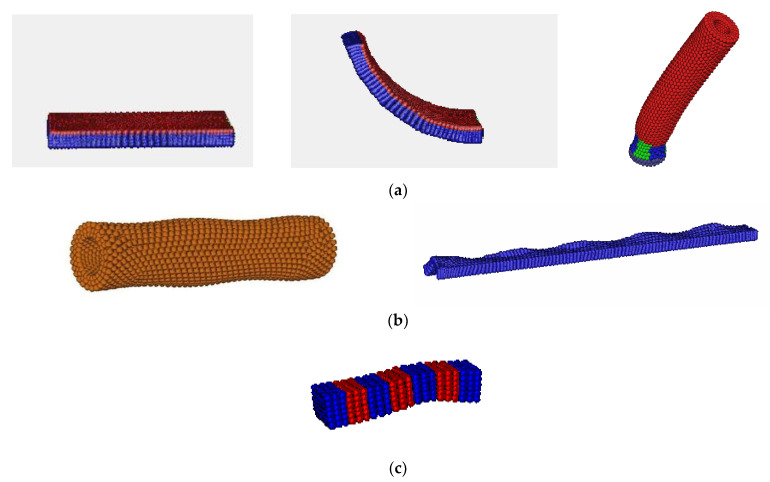
(**a**–**c**) illustrate the case studies considered in this study [26] (Reprinted with permission from [26]). (**a**) FE simulations carried out on an azo-doped nematic elastomer beam which has been anchored by its right edge. When the beam is exposed to light, it automatically bends in an upward. The purpose of this simulation was to numerically investigate an experiment carried out by Camacho-Lopez et al. [57]. Far left: the beam is shown to be completely in the nematic state. Before exposure, the director is oriented horizontally. Middle: a rapid upward bend is induced by the top (red) layer when it switches to isotropic state. Right: six nematic elastomer actuators (illustrated in blue and green) arrayed in a ring at the tube’s base, cause the formation of a red rubber tube. (**b**) FE simulations—Left: peristaltic motion a nematic elastomer tube. The motion is caused by periodic modulation of the scalar nematic order parameter along the tube’s length, e.g., by temperature or light. Note that The FE mesh nodes are represented as spheres. Right: peristaltic motion in a thin film, which is designed for covering and transporting the contents of a rigid. (**c**) FE simulations—A nematic elastomer, mimics the crawling motion of an earthworm.

Selinger et al. [26] used a Hamiltonian FEM approach to model elastodynamics of 3D devices made of nematic elastomers. Said devices included azo-doped nematic elastomer beam, peristaltic pumps and a soft self-propelled robot. They predicted that their FE based approach would be able to simulate large deformations in a variety of soft materials, such as the response behavior of said materials to changes in orientational order. Figure 7 illustrates the case studies considered in this study [26].

The models were discretized into tetrahedral meshes and mathematical formulations for potential energy and kinematic energy were subsequently used to construct the Hamiltonian. In order to derive the Hamiltonian, a method proposed by Broughton [58] was used in which, a Green–Lagrange strain tensor was used instead of linear strain tensor. This replacement allowed the algorithm to not be limited to small rotations.

Another study which investigated the deformation of thin film LCEs samples was conducted by [59]. Light was considered as a source of simulation for inducing deformation in the samples, but instead of considering the photo-strain as being isotropic, the authors considered induced photo-strains as being anisotropic and depth related. A model was developed for predicting the deformation of said thin films while considering the photo-strains to being anisotropic. Two parameters are utilized for describing the photo-strains. These parameters are related to the light intensity and its reduction, i.e., its attenuation, as it travels through the thickness of the sample. Macroscopic deformations were modeled via two equivalent representations of photo-strains namely, (a) variations exhibiting an exponential behavior through the sample thickness and (b) a two-layer model. The latter is better suited for performing FE simulations and design purposes. Moreover, a two-layer model is advantageous for FE calculations and for the design of photo-strain patterns. The analytical model was able to accurately predict the initial saddle shaped deformation of the plate shaped sample used in the authors’ study, and its subsequent nonlinear behavior along with its eventual cylindrical bending, under the influence of large photo-strains. The results were in good agreement with data from more accurate nonlinear FE models, even though the authors’ model assumed that the curvature of deformation is constant. Detailed mathematical procedures can be found in the authors’ original manuscript.

FE analysis was carried out by Mbanga et al. [27] to perform 3D elastodynamics simulations, to examine the impact of orientational domains on strain-induced instabilities in LCEs, with the objective of studying the physics which govern the dynamic behavior of nematic elastomers. The authors focused on a film of mono-domain material and studied the onset of stripe formation. The film was considered to be stretched along an axis, perpendicular to the nematic director. An advantage of the proposed FE model was that it was Hamiltonian-based and incorporated terms which coupled strain with the nematic order. This allowed for several key features such as shape evolution, mechanical response to mechanical stimuli and the associated evolution of microstructures in the sample. As in [26], instead of a linear strain tensor, a rotationally invariant Green-Lagrange strain tensor was used for deriving the Hamiltonian. The reader can consult the authors’ original manuscript for a complete mathematical description of the method.

**Figure 8:**
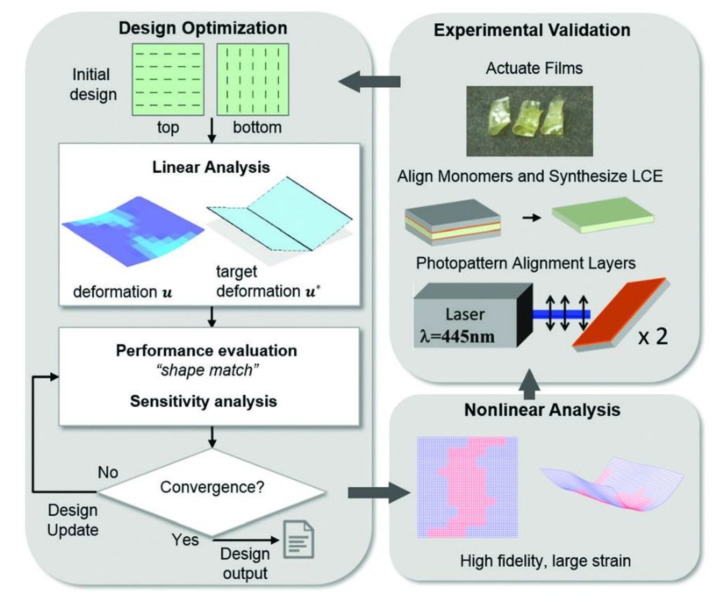
Outline of the design and experiment process outlined in [60] (Reprinted with permission from [57]).

Topology optimization was used by [60] for generating reliable folding patterns in monolithic LCEs. Order/disorder distribution and the orientation of directors where the targets of the optimization procedure to ensure that the folding patterns induced by stimulation from heat source would turn out as desired. Linear brick elements were used to perform a low fidelity FE analysis so that computational sensitivity analysis costs could be reduced. By using FEA and optimizing strain distribution, the targeted deformation of an LCE film into a hinge shape was observed. By using photoalignment, the LCE patterning technique is able to control both strain strength and orientation distribution within small domains, known as pixels, by specifying the local strain tensor within that pixel domain. Therefore, finding the optimal distribution for order parameters and director orientations, such that the final shape of the deformed shape of the film is the best possible match with the target shape, is the focus of the optimization problem. The problem is made further difficult once the ordered area fraction is taken into account. Svanberg [61] developed a MATLAB code, The MathWorks, Inc, Natick, MA, USA, based on the moving asymptotes (MMA) method. This method was chosen because of its usefulness in handling problems with multiple design variables. For evaluation of predicted designs and their folding performance, ANSYS 11.0 was used for carrying out nonlinear analysis under large strains. After performing the numerical analysis, for both the small and large strain regimes, the obtained director orientation and order profiles were used as input for fabrication of experimental samples. Once the samples were prepared, they were tested using a uniform heating method. The flowchart in Figure 8 demonstrates the design and experiment process.

The results show that some of the assumptions made in the model, e.g., the multi-stability in patterned films and the undesired deformation in polydomain, differed from what was observed from the experimental results. The authors reported that incorporation of said effects into the model would increase the predictability of complicated deformation and repeatability in experiments, but would lead to more computation costs. Therefore, based on the desired level of accuracy, an appropriate treatment method must be determined for treating of the said complex phenomena. Moreover, they further stated that the detail level of the model used in their study, was sufficient for their desired level of accuracy and that another possible method would be to augment the experimental procedure (when dealing with complex target shapes) in order to reduce the undesirable observed behavior of the material and the computation costs. The authors stated that potential modification to the method would include (a) enforcing symmetry in optimal design, (b) utilizing design filters to implement smooth transitions for the director orientation, and (c) using parallel computation for the FEM computations.

More recently, Konya et al. [23] demonstrated that including effective bending energy into formulations used for simulating elastodynamics behavior of elastomers is necessary for obtaining accurate predictions regarding behavior of 3D devices stimulated by heat. The method used for carrying out the FEM analysis was comprised of discretizing the domain into tetrahedral meshes and using the Hamiltonian. Ultimately, the author’s demonstrated the effectiveness of their methodology to the prediction of LCE device behavior where defects, namely azimuthal defects (+1, −2, and −4), connecting two straight (non-LCE) elastic side walls with an array of short LCE beams and incorporating two different director domains to each end of a beam mounted on a substrate, at one of its ends. The authors concluded that their model could play a helpful role in modeling bending behavior of samples with non-uniform nematic directors. Utilizing patterned LCEs for device fabrication is somewhat limiting due to the fact that the nematic director field may only be controlled via manipulating anchoring patterns at the sample’s surface. Therefore, an arbitrary 3D director field could not be realized [33].

By conducting 3D FE simulation studies, Gimenez-Pinto et al. [62] studied director-encoded chiral shape actuation, in thin-film nematic polymer networks under external stimulus, namely via temperature. Moreover, the authors designed an auto-origami box, which had an initial flat shape, corresponding to an initial nematic state, and could bend in one of four well-controlled bend bending patterns, based on the corresponding order changes. Hybrid particle FE elastodynamics simulations were used for designing the said actuator. Once again, as in many previous studies, tetrahedral meshes were used for discretization of the actuator domain and a Hamiltonian, based on the energy, both kinetic and potential, is derived for the system and related to the nematic director field using mathematical formulations described in detail in the authors’ original manuscript. The results showed that, imprinting non-uniform liquid crystal director fields in thin-film polymers could lead to shape transitions which were responsive to external stimuli. Furthermore, such an encoding would depend on geometry of the director, aspect ratio and temperature of the sample. The results obtained for elastomer ribbons, twisting of crossed-shape, self-folding actuators, and auto-origami box show good agreement with experimental and analytical results [63,64], demonstrating that the hybrid particle FE elastodynamics method is very suitable for studying the deformation behavior of LCE’s, with a non-uniform nematic director, in 3D.

Neufeld et al. [65] used a hyper-elastic solid mechanical model, previously presented in [66,67], for simulating the mechanical response of a nematic liquid crystal network (LCN) to temperature variations. This was achieved by knowing the texture of the nematic director field, which was imprinted during cross-linking process and with measurements done on the mechanical material parameters, performed on uniformly-aligned LCN samples. A compressibility method in the hyper-elastic model, was proposed by decomposing the experimentally measured contributions to deformation of uniform-aligned LCN samples, into two categories, namely isotropic and anisotropic. The hyper-elastic model was solved using mixed FEM. An open-source FE software, FEniCS project, version 1.5, developer “Chalmers University of Technology, Sweden” [68], was used for implementing the proposed method. For model validation, data from experiments carried out on LCN cantilevers, with hybrid aligned nematic texture, were used. Furthermore, the performance of an LCN multi-legged gripper design was analysed and optimized to serve as a proof-of-concept for the model. The results showed a significant promise for the simulation-based design of LCN materials, with an eye on overcoming the inherent complexity and cost involved in designing such devices.

Cirak et al. [69] used a FE code to study the energetics of LCEs, when stress is utilized as a stimulant for reorienting the nematic directors. The authors stated their mission as: (a) establishing that via manipulation of soft deformation behavior, changes in the Gaussian curvature of the samples could be created using little energy; (b) developing a numerical method that could accurately study the large deformations in nonlinear samples. The FE method was used to discretize the energy functional (using box-spline functions). An overview of the development process of the functional itself is as follows. The Verwey–Warner–Terentjev (VWT) [70] energy description, proposed by [49], is first considered. Then, the authors [69] demonstrated that the elastic energy could be decomposed into two separate terms, one constituting the deformation of the nematic director and the other, representing the elastic component. This decomposed form of energy functional is then applied to membranes having lengths much larger than their thickness. Finally, the method was used to simulate the stress induced deformation of thin LCE membranes, and it was shown that physically meaningful deformations could be attained, even when the energy functionals were not convex.

Another application of FE models is the study of acoustic properties of LC nematic elastomers (LCNEs). Many aspects of acoustic properties have been previously studied by various researchers including: (a) propagation of acoustic wave through elastic LCNEs, done by Terentjev et al. [71,72]; (b) spectral and polarization characteristics of acoustic waves propagation, in low-frequency limit [73]; (c) reflection features of homogeneous elastic waves from free surface of LCNE [74]; (d) characteristics of surface and edge waves in solids with nematic coating [75]; (e) exotic properties of Rayleigh waves in LCNEs [76], and more recently, (f) wave dispersion in LCNE phononic crystals have been studied by [77].

An energy function method was proposed by Shuai et al. [78] for modeling deformations induced by heat in nematic elastomers with periodic structures. In the proposed method, band variation in LCNEs’ porous phononic crystals were extensively investigated. The numerical simulations were carried out by developing a model in COMSOL software, COMSOL Inc., Burlington, MA, USA, using triangular Lagrange quadratic elements, and importing the built model to MATLAB, The MathWorks, Inc, Natick, MA, USA, for implementation of the developed numerical algorithm. The authors’ method consisted of first, conducting a quasi-static analysis to show the periodic variation of LCNE porous phononic crystals, due to deformation of LCNE matrix, caused by variations in temperature. Subsequently, wave propagation analysis was performed according to the deformed lattice, followed by a discussion on the influence of porosity, temperature, and director orientation on the absolute band gaps (ABGs). A complete description of the authors’ methodology can be found in the original manuscript.

## 4. Monte Carlo Simulations

By reduction of the length scale of the problems, scenarios may arise where the constitutive relations based on the assumptions of continuum medium are no more valid. Therefore, in microscale modeling problems, methods such as Monte Carlo (MC) are commonly used [32]. Monte Carlo molecular simulation is the application of Monte Carlo modeling approach for modeling the molecular behavior. This method is based on the equilibrium statistical mechanics, which typically relies on statistical methods, probability theory and microscopic physical laws [79]. MC produces states based on Boltzmann probabilities, without the reproduction of the dynamic of the system [32]. In order for the demonstration of the probable distributions of the micro-particles matching with the state of the system, in the MC simulation method, the molecules are supposed to move randomly [80]. Due to the randomness of the movements of the micro-particles, the pattern of their movements cannot be produced, meaning that these particles do not follow a physical trajectory, so this feature makes the MC method be a good option for simulation of the systems which are in thermodynamic equilibrium state [80]. This method is capable of demonstrating any possible connection between macroscopic changes and microscopic parameters.

Molecular simulations can also be an important tool for investigation of LCEs. The main aim of this simulation is to correlate between molecular structures, on a microscopic scale, and macroscopic properties of LC phases. To achieve this goal, in different studies, researchers have made certain assumptions for the molecular structure, and based on those assumptions, determined the large-scale order of the resulting system as a function of several variables such as temperature and applied field, by simulating a large number of interacting molecules. This method is not only a good tool for studying phase changes in LCs and their structural texture, but also, is helpful for developing new LC systems for various applications.

Most of the studies done on the macroscopic level, involve an initial assumption for the structure of the LC molecules. Afterwards, using intermolecular potential, the molecules are allowed to interact with one another [81]. This approach has both advantage and disadvantages. The advantage is that it provides an opportunity to simulate experimental samples and show how small variations in the chemical structure can affect the material properties on a macroscopic scale. On the other hand, the disadvantage is that this approach can only be implemented for relatively small systems, due to it being computationally intensive. This could lead to a shortcoming in the method’s ability to model all of the important characteristics of LC ordering. The accuracy of classical potential functions which are readily used can also be an issue. The solution devised to combat these hurdles, adopted by many researchers, has been to use simplified models for simulating molecular structures and interactions [82].

MC method has also been used for performing large-scale computer simulations for studying the effect of electric field as an actuation tool, to clarify the operational behavior of the LCEs on a molecular level [83]. Skačeja et al. [83] used a semisoft deformation MC method [84] whereby the applied external field was perpendicular to the nematic director, and with a mode that alternated between the orientationally disordered isotropic and the aligned nematic phases. In this study, all liquid crystal molecules were represented by uniaxial ellipsoids and the total interaction energy was calculated by summing the non-bonded and bonded intermolecular contributions, using Soft-Core GB Interaction, with the interaction energy of the external field. A complete description of the authors’ [83] methods can be found in the original manuscript. The results illustrated that for the first mode that increasing the applied field strength resulted in a response in the presence of monomers. Furthermore, in the latter case, higher switching thresholds were observed to happen compared to typical thresholds for semisoft actuation mode involving director rotation.

Pasini et al. [85] proposed a simple coarse-grained lattice model for LCEs, undergoing uniaxial strain, and by employing large scale MC simulations, showed that the model was able to reproduce results obtained from a variety of experiments including stress strain, order, light transmission and thermal effects. Materials with both homogeneous and inhomogeneous cross links were considered by the authors. The proposed model was developed based on three main theoretical building blocks, viz. polymer network exhibits rubber elastic behavior, Vander Waals-type, or steric type interactions between nematogenic units and coupling between stress and alignment within the samples. A complete description of the model can be found in the authors’ original manuscript. By obtaining the total Hamiltonian for LCE via summation of individual Hamiltonians obtained for each of the individual aforementioned building blocks, and through conducting constant-force MC simulations the authors were able to illustrate how the model was able to accurately reproduce experimental results. However, it was also stated that, due to the model’s inability to capture soft elasticity behavior, it is unable to treat shear deformation.

Scajek et al. [86] also developed a coarse-grain model for studying LCEs, which underwent biaxial strain. The developed model was capable of predicting calorimetry data and deuterium magnetic resonance spectra. Using MC simulations, whose main building blocks are the same as [85], whereby the sum of pseudo-Hamiltonians describing rubber elasticity, anisotropic interactions between biaxial mesogenic units, and a newly developed pseudo-Hamiltonian for coupling orientational ordering to strain (adapted from the approach developed by Straley [87]), are used to model monodomain biaxial liquid crystal elastomers (a thorough description can be found in the authors’ original manuscript).

**Figure 9:**
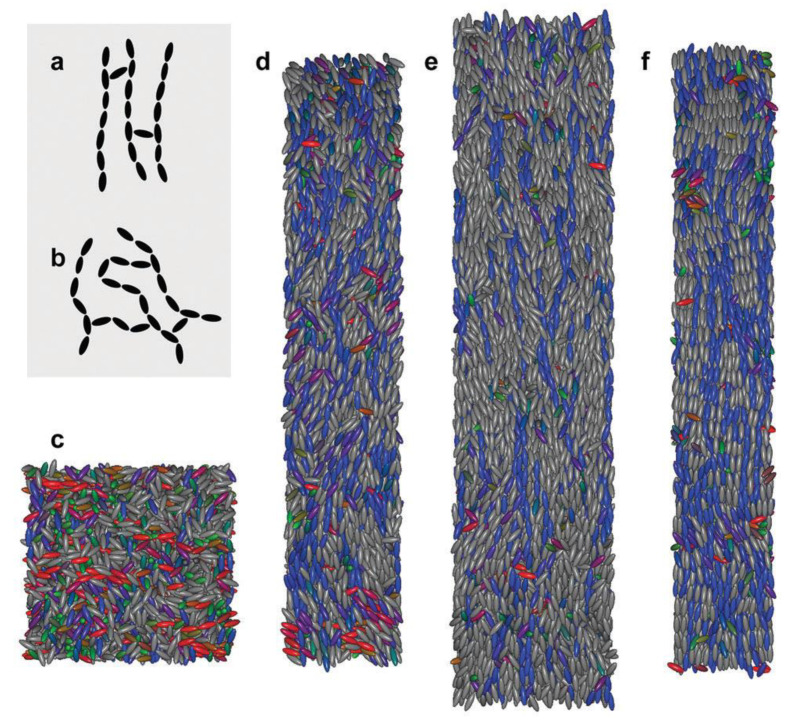
(**a**,**b**) illustrate the regular and irregular samples considered by [88]. (**c**–**f**) show the isotropic, nematic (less swelling), nematic (more swelling) and smectic phases, respectively (Reprinted with permission from [88]).

Scajek [88] has recently used MC simulations to study how sample preparation affects the nematic-isotropic behavior in LCE samples with both regular and irregular polymer networks. A full description on how these two sample types can be created can be found in [7,28]. Main-chain systems were considered due to their extreme strain alignment coupling. Soft-core GB potential [89] was used for the simulations. Uniaxial soft-core GB ellipsoids were utilized for modeling mesogenic molecules and for assembling LCE networks, and also to illustrate non-bonded swelling monomers. Figure 9 illustrates the schematic representation of samples considered in the study.

The results reported by the author [88] showed that the nematic-isotropic (NI) transition could be shifted to higher temperatures by the presence of a stabilizing polymer network, that provides an internal orientational easy axis. Moreover, as the imprinted orientational easy axis becomes more pronounced, the said shift will become larger in regular samples.

**Figure 10:**
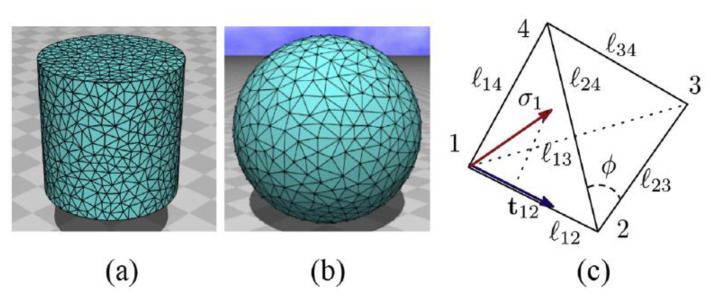
(**a**,**b**) represent both cylindrical and spherical domains. (**c**) shows how the discrete Hamiltonian is defined on each tetrahedron [90] (Reprinted with permission from [90]).

Koibuchi et al. [90] studied 3D LCE in the context of Finsler geometry (FG) [91,92]. The purpose of this was to gain a better understanding of the anisotropic phenomena exhibited by LCEs (interplay between LC and polymer parts which give rise to soft elasticity behavior) by introducing a variable, σ, which represents the directional degrees of freedom of a LC molecule, located at a 3D position r, so that the interactions between LC molecules and bulk polymers could be understood and simulated. Such a parameter was absent in previous coarse grain studies such as [85,86], where the net alignment of polymers is only considered in the derivation of the pseudo-Hamiltonians [85,86]. By performing MC simulations on a cylindrical body, placed between two parallel plates, the authors [90] found that the tensile stress and strain are in good agreement with reported experimental results [93,94]. Furthermore, the elongation of a spherical body, having free boundaries, is also observed in the data obtained from the MC simulations, and they were found to be consistent with previously reported experimental data [93,95]. Figure 10 shows the cylindrical and spherical geometries developed by implementing the discrete FG model utilized by the authors. The authors [90] introduced a discrete FG model for a 3D LCE, which is defined using the technique presented in Ref. [96]. A cylindrical body and a spherical body in R3 are constructed by the Voronoi tessellation (Figure 10a,b) with tetrahedrons (Figure 10c), which are composed of vertices, bonds, and triangles. Based on their results, the authors offer speculative insights into applications of FG modeling in comparison to other frequently used methodologies.

- The mechanism of a deformation of a thin LCE, under non-uniform illumination of visible light, can be understood in the framework of FG modeling. For such thin LCE, the temperature dependence of physical quantities can be evaluated.
- The second is the deformation of LCE under external electric fields. The variables σ is aligned, either along or vertical to an electric field, and deformation of LCE is expected to be independent of how σ is aligned. Finally, the J-shaped stress–strain diagram of biological materials, e.g., blood vessels and skin, can also be considered in the scope of FG modeling.

Shape and volume phase transitions of LCEs submerged in a solvent was analyzed by Egorov et al. [97] in the context of the Finsler geometry (FG) model, which was proposed by [98] for investigating the behavior of liquid crystal elastomers exposed to electrical fields. In this study, they expand this FG model by adding an Ising-like variable to account for both swollen and nonswollen LCE states. The three-dimensional body is discretized using tetrahedrons to describe the metric tensor and discrete Hamiltonian. Two variables, σ which defines as shape anisotropy of LCEs and τ representing swollen and nonswollen states of LCEs have been applied to the vertex of tetrahedron. According to the results obtained from Monte Carlo simulation study, it is demonstrated that the model effectively replicates the variations in the swelling degree along with the smooth change of the shape anisotropy affected by increasing the temperature.

## 5. Molecular Dynamics Simulations

Molecular dynamics (MD) simulation is a numerical method used for the study of the interatomic or molecular movements by considering the interaction between these particles. The movements of a complex system containing molecules or atoms are determined by the Newton’s motion laws (for spherical particles) or Newton-Euler (for non-spherical particles) [80,99]. The forces and potential energy between two atoms and molecules can be calculated by the use of mechanic force field and interatomic potential, which relies on the position of each particle in space [100]. This method offers a variety of benefits in terms of atomic and molecular simulations, in fact, it provides the opportunity of examining the dynamics of particles which cannot be easily observed through direct way. This method is appropriate for both equilibrium and time dependent studies [80,100]. Since MD is a deterministic method, the trajectories of molecules can be determined, so it leads this method to be a good option for understanding the interactions between small molecules [80]. The main drawback that can be addressed in this method is its high computational cost of modeling the behavior of molecules.

MD simulations have been extensively used for reproducing principal features of LCEs with side-chain architectures. An algorithm for achieving uniform spatial distribution of crosslinks, during melting, was developed by [101]. The elastomer is formed by crosslinking of the melt in the smectic A phase. After reaching equilibrium and subsequent isotropic smectic transition, the memory effects were studied. In addition, the effects of applying a uniaxial load, both before and after the sample had gone through smectic–isotropic transition, were studied.

Furthermore, it was reported that a number of experimental observations were numerically reproduced, of which include:

1. Crosslinking led to a 5 °C increase in the smectic–isotropic temperature.
2. In a creep experiment, elastomers in the isotropic phase were observed to have high elasticity.
3. When the elastomer undergoes the smectic–isotropic transition, memory effects in LC sample were observed.

The authors stated that, the main goal of this type of simulation was to predict how the chemical structure of these materials, influence their mechanical behavior [101]. They also reported that the simulation capabilities could be further enhanced, to consider larger system. Therefore, it would be a feasible approach to consider: (a) conducting MD simulations using moderately coarse-grained molecular models, (b) include the impacts of finite system size, and (c) incorporate continuum mechanics theories.

MD simulations were used to study how phase transition from polydomain to monodomain occurs in LCEs on a molecular scale, using a coarse grain model in which ellipsoidal shapes were used to describe the mesogen particles [8].

**Figure 11:**
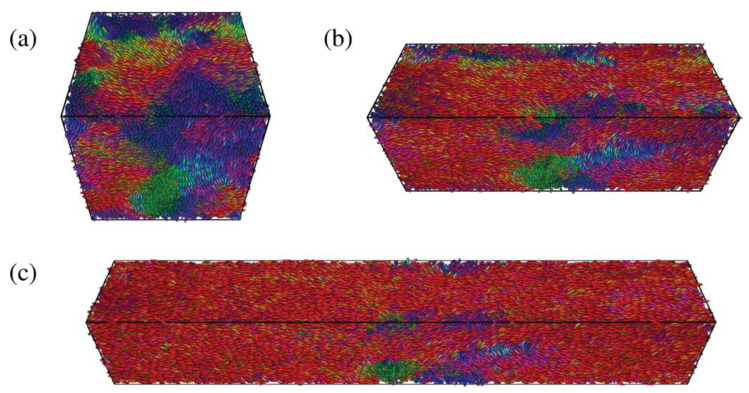
Results showing the configuration of LCEs, having rigid cross-linkers, at different stages, during a simulation where the strain-rate is kept constant. (**a**) Initial state, showing a clear polydomain structure. (**b**) Alignment begins after applying strain. (**c**) As the strain is further increased, only small misaligned clusters remain [8] (Reprinted with permission from [8]).

The authors’ developed molecular model could describe the polydomain state and consisted of a side chain liquid crystal elastomer (SCLCE). The SCLCE had rigid cross-linkers, and the interaction of said cross-linkers were tuned, such that local ordering to the easy axis of the cross-linker was promoted. A complete description of the model along with its details are presented in the authors’ original manuscript. GB potential was used to describe the interactions between mesogens. Figure 11 illustrates the configuration of LCEs, at various stages of applying external stress.

In conclusion, the authors reported that their simulations provided direct evidence for the effects of stress on polydomain material namely, that the domains rotate and merge, leading to macroscopic deformations. Moreover, the results obtained via MD simulations are in agreement with experiments conducted by [102] and support the conclusion that, domain rotation leads to the formation of stress plateaus in LCEs.

**Figure 12:**
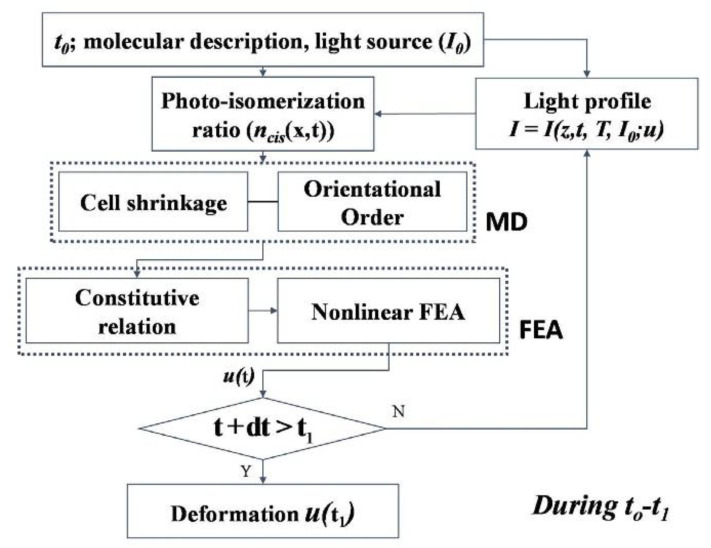
Workflow of the multiscale model. At an instance in time (t), a photoisomerization ratio is calculated for given values of light intensity (I_o_) and temperature (T); and then is used for carrying out MD simulations, which in turn will provide microscopic information to the nonlinear FEA [103] (Reprinted with permission from [103]).

Chung et al. [103] presented a newly developed multiscale framework for studying the photomechanical behavior of photo-responsive polymer networks (PRPNs). Regarding the photomechanical behavior from the viewpoint of *trans*-to-*cis* photoisomerization, azobenzene-based acrylate side-chain PRPNs were the focus of this study [104]. The developed method is comprised of two sections:

(i) MD simulations are utilized to examine the small-scale behavior of the structure. The MD method used is based on the work of Choi et al. [105]. It integrates energetic relaxation with multi-step crosslinking. To fully consider the effect of kinked *cis-* molecules, a heuristic equation [106], which substitutes Landau expansion for Maier–Saupe phase transition [6], has been modified parametrically and subsequently been used for carrying out the simulations.

(ii) Due to the failure of the macroscopic viewpoint in capturing key microscopic information, extracting the modulated thermomechanical phase behaviors, which represents variations in the long-range mesogenic interactions when short-range atomic interactions are present, was achieved by a sequential multiscale framework. In this framework, microstate-related parameters become upscaled and related to macroscopic deformations. Figure 12 shows how the multiscale framework is implemented. This algorithm upscales the microstate variations found in MD simulations, and are subsequently utilized to simulate macro-scale deformations exposed to a determined molecular composition temperature *T* and intensity of the light source *I_o_*.

**Figure 13:**
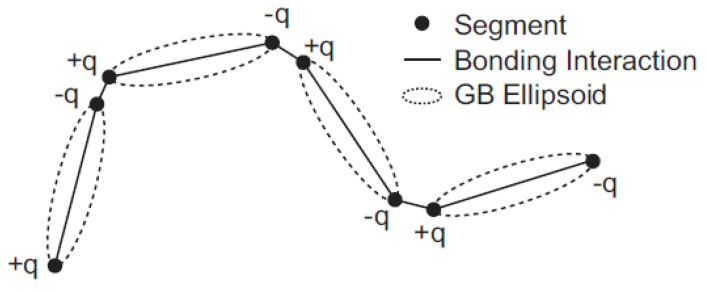
Schematic of the coarse-grained LCP model proposed by [107] (Reprinted with permission from [107]).

A coarse-grained model consisting of mesogenic molecules and smeared charges for the study of liquid crystal polymers (LCPs) was developed by Tagashira et al. [107]. The coarse-grained LCP model can be thought of as a modified version of the model proposed by Skaceja et al. [83,89] where the intermolecular interaction between ellipsoids is modelled using the soft-core Gay-Berne (SCGB) potential. However, the major difference from the initial model is that point charges have been introduced in order to enable direct calculation of the piezoelectric tensor. For the smearing function of the electrostatic force, Gaussian error function was developed to calculate the Coulomb force between tow point charges Q and q. For implementation of the model, a course-grained MD program COGNAC (COarse-Grained molecular dynamics program by NAgoya Cooperation, Nagoya, Japan) released in 2002, Japan [108,109], was used. Figure 13 shows the coarse-grained LCP model used by the authors [107]. MD simulation system is a cubic cell of volume V, consisting of 121 LCP molecules. Each molecule is comprised of 30 GB particles and 3630 GB monomer particles. Thus, the LCP model with smeared charges exhibits both properties of liquid crystals and polymers. The authors reported that their model succeeded in simulating LCP reaction to electric field, and stated that it can be used to predict mechanical characteristics of LCPs arising from the alignment of the mesogenic units.

In case of Molecular Dynamics (MD) simulation studies, the force field, which determines the inter- and intramolecular interaction, is critical in providing the precise time and length scales correlated with molecular processes that define the macroscopic behavior of the materials. In this regard, in a recent work by Prathumrat et al. [110], a molecular simulation-based study was developed to discuss which force field can better define the behavior of typical LCE materials. The critical features of LCE material, such as steady-state density, transition temperature, and viscoelastic properties, were investigated through MD simulation and experimental study. Three force fields, namely, Dreiding, PCFF, and SciPCFF, were separately applied to the LCE modeling system. They found that the thermomechanical properties of the system modeled by the SciPCFF potential are well consistent with the relevant experimental data. As the next step, using this force field, they tried to predict the shape memory properties of this material. The equilibrium state of the total energy at each shape memory step confirmed the suitability of SciPCFF force field in liquid crystal elastomer modeling.

## 6. Other Numerical Methods

**Figure 14:**
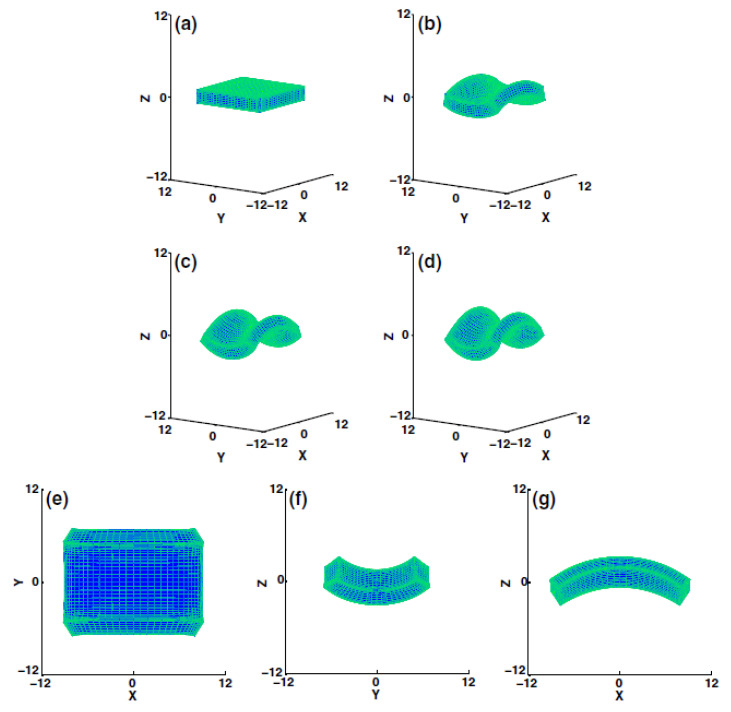
The shape evolution of the LCE when inhomogeneous temperature distribution is applied. (**a**) the LCE is in its initial state (**b**,**c**) both show two transitional states, and (**d**) demonstrates the sample after reaching steady state condition. (**e**–**g**) illustrate the LCE sample of (**d**) from three different points of view [112] (Reprinted with permission from [112]).

**Figure 15:**
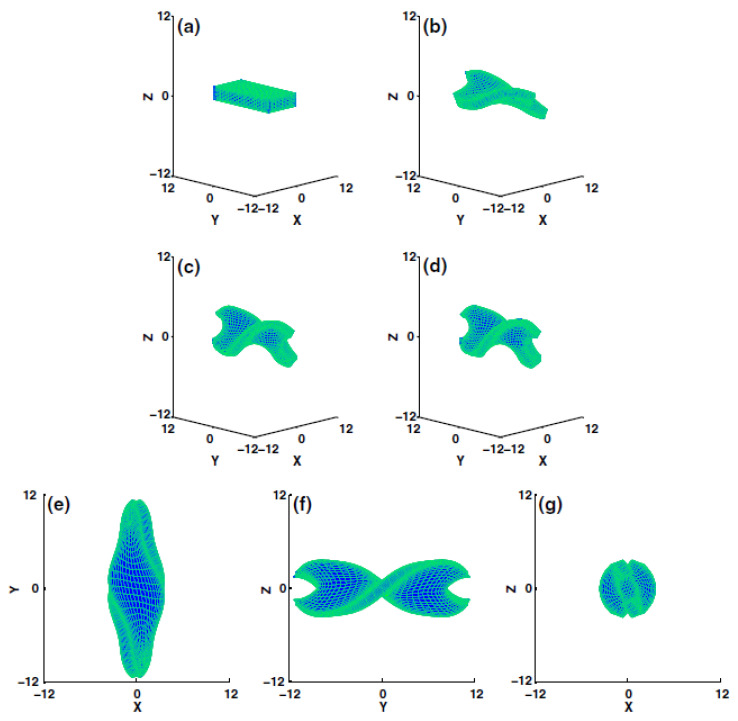
Evolution of the LCE sample as a result of inhomogeneous temperature distribution. (**a**) the LCE is in its initial state (**b**,**c**) both show two transitional states, and (**d**) demonstrates the sample after reaching steady state condition. (**e**–**g**) illustrate the LCE sample of (**d**) from three different points of view [112]. (Reprinted with permission from [112]).

Zue et al. [111] developed a non-local continuum model to study the dynamic behavior of LCEs, that was based on the work of [112], by using a novel preconditioner based on Chebyshev spectral collocation method, which was validated using experimental observations. In these formulations x *(α, t)* is the location of a material at point *α* in an LCE sample at time *t* which must be found under the applied external stimuli and relevant boundary conditions. Therefore, solving the developed continuum model for x is the objective. When solving the functional expressions for LCE, highly nonlinear terms, developed in a Lagrangian frame, must be accounted for; this, is where the Chebyshev spectral method is utilized along with implicit-explicit scheme (IMEX), which is a hybrid of the second-order Adams–Bashforth scheme for the explicit terms and the Crank–Nicolson scheme for the implicit terms [113,114] and [115]. All the examples considered in this work involved the study of illumination on LCE deformation using the GMRES method [116]. Figure 14 and Figure 15 illustrate the modeling results for LCE deformation due to inhomogeneous temperature distribution profiles within LCE samples.

In this experiment, a linear temperature drop occurs from the top till the bottom of the LCE sample while being uniformly distributed on each horizontal cross section. During the evolution process, the spatial distribution of temperature is maintained.

**Figure 16:**
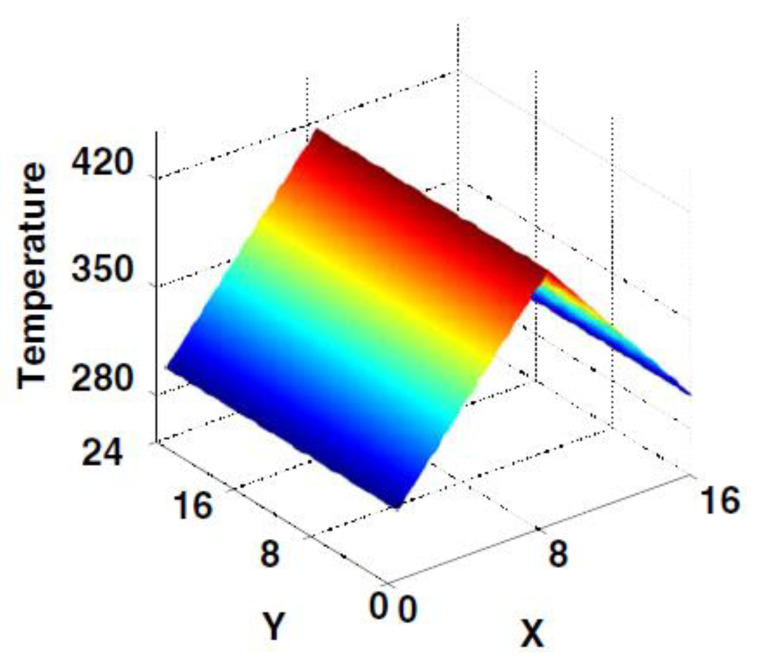
Temperature distribution applied to the sample in Figure 15 [111] (Reprinted with permission from [111]).

In this experiment, the temperature along the path from the top to the bottom of the sample is constant. The horizontal temperature distribution is shown in Figure 16. During the evolution process, the spatial distribution of temperature is maintained. This simulation illustrates the shape changing feature of the material.

Thermomechanical models are models where the strain is analytically described in terms of variations of temperature. A commonality among various thermomechanical models is that they apply a constant temperature increase, usually induced by changes in the temperature of the environment [117]. Examples of thermomechanical models can be found in the works of [118,119]. A thermomechanical model was proposed by Jin et al. [36] to model thermal–order–mechanical coupling behaviors in LCEs. The anisotropy of LCEs was described using a metric tensor. It was assumed that the total energy was a function of deformation gradient, direction of the director, order parameter and biaxiality. After deriving equations for mechanical and phase equilibrium, total free energy was considered to be a sum of the entropy-induced elastic energy and the Landau–de Gennes nematic energy. Said assumptions were used to derive the Cauchy stress–deformation relation and the order–mechanical coupling equations. With these equations, different thermal–order–mechanical behaviors of LCEs could be studied, e.g., how prolate LCEs behaved, in different directions, under the influence of stretching, compression, or simple shear force. A complete description of the mathematical formulation can be found in the authors’ original manuscript.

A soft matter cell model based on Lagrange-type mesh-free Galerkin formulation and subsequently related algorithms was developed by Zeng et al. [120] in order to model the mechanical procedure by which biological cells exchange information. In this paper, the cells were modeled as LCEs. The modified Mooney–Rivlin material [121] was adopted to model the extracellular matrix, the bulk energy density of an incompressible nematic elastomer was modeled using formulation developed by [122] and models developed by [123] were rewritten and modified to account for the cell adhesion and motility. The simulations were performed for 3D case studies. The soft matter cell model developed in [120] was limited in scope and failed to consider chemo-mechanical interactions such as the actin-myosin dynamics. Moreover, the effects of fiber contraction and membrane protrusion during cell spreading, due to active stress, have not been considered. Therefore, the model is more suitable for studying the overall mechanical behavior of cells at early stages of cell spreading. Finally, the numerical results were compared with experimental data for validation. The numerical data showed that, during spreading, cell motion includes gliding and rolling forward along the substrate.

A coupled electromechanical model was proposed by Cohen et al. [124] for the studying the mechanical behavior of Smectic-A LCEs, in response to electromechanical stimuli. In this model, a free energy density function is obtained by summing contributions from: (a) elastic free energy [38], (b) change in the layer thickness [125,126], (c) variation in chirality, or equivalently the tilt (or rotation) of the director [125], (d) energetic contribution of the electrical sources to the total energy [127], and (e) external mechanical loading. Once the total free energy function has been obtained, it is solved numerically for three different case studies were, first, electricity induces deformation in a sample, second, deformation induces polarization in the LCE sample’s mesogens, and third, the electromechanical response of a cylindrical smectic-A elastomer with helical layers was investigated and it was demonstrated that upon usage of an electric field, the pitch and the helix angle control the macroscopic behavior. Finally, the authors reported that the numerical results were qualitatively in agreement with experimental data.

More recently, Keip and Nadgir [128] proposed a continuum mechanics, phase-field method for modeling the electro-elastic behavior of nematic LCEs. The nemato-electro-elastic model is coupled with the Landau–de-Gennes theory and the phase field is described by using a tensorial order parameter. The model first assumes that the strains are small and afterwards, implements the phase-field formulation using FEM, to investigate the evolution of microstructures in nematic elastomers, under the influence of various boundary conditions. This model contrasts with its predecessors [129,130] in that, it considers the full Landau–de-Gennes order parameter as phase field; therefore, allowing the new model to overcome the numerical challenges inherent in other director models [131,132]. A complete derivation of the mathematical framework used by the authors [128] can be found in the original manuscript. The authors’ results demonstrate that the proposed model was able to characterize how the nematic microstructures evolved to a good degree.

Cui et al. [133] presented a transient analytical solution for studying the thermos-mechanical behavior of a soft robot, consisting of a strip of LCE and an accompanying inert polymer, actuated by an ultra-thin open mesh heater. The model consisted of a one-dimensional heat transfer equation coupled with a bilayer beam model. The heat transfer equation was solved using Green’s function method and the results were used in the bilayer model. The results obtained from the analytical model were validated with data from FE simulations carried out with ABAQUS software. The authors concluded that the proposed model was able to accurately predict the thermo-mechanical behavior of a soft robot undergoing Joule heating.

A continuum mechanics model was developed by [134] to serve as a general framework for studying the interactions between LCs and polymer backbones that constitute an LCE. The model was able to take into account, large deformations as well as large director deformations. The novelty of the proposed model was that in addition to taking into account the director or order tensors, along with their respective time derivatives, the deformation gradient and its derivative with respect to time was also incorporated into the model. The dissipation principle was used as a basis for deriving the momentum balance equations, using the Cauchy stress tensor. This tensor included the contributions from bulk elasticity as well as changes in orientational order. Finally, a general form of the free energy density and Rayleigh dissipation were proposed along with a simplified model for studying the reorientation of thin film LCEs, induced via stretching.

## 7. Discussion and Summary Remarks

LCEs are a class of synthetic materials with specific features that make them appropriate for use in many applications. They combine the elasticity of polymers with the liquid crystalline properties. Their behavior to external stimuli has made them a not only a hot topic of research into their mechanical behavior, but also as a strong candidate for various applications. In respect to their development, it should be noted that based on the recent researches, numerous fabrication methods for LCEs with controlled mesoscale structures in two and three dimensions have recently been implemented [135,136]. Owing to the potential of these fabrication techniques, there is a growing demand for simulation approaches that can accurately model LCEs physicochemical and mechanical behavior with the aim of approaching the desired design [53]. In this regard, it is expected that the role of mathematical modeling and numerical simulation studies in LCE design and development will be highlighted.

In this study, various numerical tools for studying and predicting the behavior of LCEs have been reviewed with the aim of (a) offering categorization of the main numerical strategies used by researchers, (b) providing examples of how they have been utilized, and (c) providing a comprehensive guide for future researchers on the specifics involved with each introduced method. In keeping with the said aims of this study, Table 1 and Table 2 are presented with the aim of: (a) providing a more categorical view of the material reviewed in this work, and (b) highlighting their methodology and formulation, respectively, so that readers can have a categorized catalogue of LCE numerical modeling research. Note that, for the sake of brevity, not all of the reviewed papers have been categorized in the following tables.

The tabularized findings in Table 1 and Table 2 have been summarized, in order to provide a concise set of recommendations, to act as reference for researchers:

- **FEM**: Usable for macroscale problems for solving a set of partial differential equations based on the consideration of the continuum medium. A good option for analysis of macroscopic behavior of LCEs, mechanical deformation, bending, and stretching when exposed to external stimuli for macroscale actuators and devices such as beams, pumps, and modules with locomotive motion. Providing an opportunity of monitoring and optimizing their performance whenever needed.
- **MC**: Creating a connection between molecular structures, microscopic scale and macroscopic properties of LCE phases. Developing the simulation studies in a large time and space scale in comparison to MD. As this method is not deterministic, this feature makes it a good option for simulation of the systems which are in thermodynamic equilibrium state.
- **MD**: Capability of demonstration of how small changes at molecular scale alter the macroscopic features. A good choice for both equilibrium and time dependent studies. Since this is a deterministic method, the trajectories of molecules can be determined, so it leads this method to be a good option for understanding the interactions between small molecules. The main drawback that can be ascribed to this method is its high computational cost of modeling the behavior of molecules.

## 8. Conclusions

LCEs are considered unusual materials with the anisotropic properties of liquid crystal molecules and elastic and stretchable behavior of long chain polymers in common. LCEs can be made with a variety of processing methods, resulting in a wide range of tailorable and programmable properties for various applications. The dynamic nature of stimuli-responsive LCEs makes them ideal for use as biomaterials to complement or substitute the dynamic behavior of natural tissues. Based on these facts, and according to their emerging role particularly in biomedical applications, e.g., bioelectronics, cell culture, and orthopedic applications, the need for a powerful and precise tool in studying LCEs features is sensible. Thanks to the expansion of mathematical modeling methods and numerical simulation strategies, different case studies with different levels of complexity can successfully be investigated. The possibility of developing modeling tools for LCE optimization in support of experimental studies is advantageous and appealing. This paper gives a summary of the liquid crystal elastomer (LCE) modeling approaches. The aim of the review is to aid in the selection of appropriate modeling approaches for different applications. To that end, various modeling strategies are presented and investigated in terms of their merits and shortcomings. Such studies can significantly reduce the time and cost associated with time-consuming experimental trials. Through the use of such tools, having a deeper and more precise view into LCE structures and their features, even at a molecular level, would not be far-fetched.

## Figures and Tables

**Table 1:** Categorization of the reviewed numerical methods.

Method	Applications
FEM (software)	(1) studying the Quasi-soft opto-mechanical behavior of a two dimensional (2D) rectangular beam using commercial software, ABAQUS [34] (2) studying the appearance of sub-stripe patterns in sheets of nematic elastomers using COMSOL Multiphysics [37] (3) simulating cooling and stretching experiments to investigate the transitional behavior of nematic elastomers using COMSOL Multiphysics [38] (4) bridging the gap between elastomer physics and device engineering and design in LCE-NTs using ANSYS [41] (5) accurate modeling of non-uniform deformation and instability of a constrained LCE beam, used as a microfluidic valves, in addition to studying a nanoscale poly styrene (PS) film laminated piece of LCE using ABAQUS [25] (6) simulating peristaltic crawling locomotion of earthworms using COMSOL Multiphysics [43] (7) simulating the domain evolution of a square LCE sample clamped on one side and free on all other three sides, performed using FEAP software [24] (8) wrinkling behavior in sheets of stretched nematic elastomer using ABAQUS [45] (9) elastodynamics simulations on dual-phase elastic solids using SALOME [52] (10) evaluation of mechanical reaction of LCE based elements to thermal stimuli using FEM software [53]
FEM (in house codes)	(1) accurately modeling fine-scale oscillating behavior of nematic elastomers [56] (2) modeling elastodynamics of 3D devices made of nematic elastomers, including azo-doped nematic elastomer beam, peristaltic pumps and soft self-propelled robots [26] (3) studying the physics which govern the dynamic behavior of nematic elastomers by performing 3D elastodynamics simulations [27] (4) Topology optimization for generating reliable folding patterns in monolithic LCEs [60] (5) evaluation of the folding performance of various LCE sample designs [61] (6) studying director-encoded chiral shape actuation in thin-film nematic polymer networks, under the influence of external stimuli, namely via temperature [62] (7) studying the energetics of LCEs, when stress is utilized as a stimulant for reorienting the nematic directors [69] (8) analyzing and optimizing the performance of an LCN multi-legged gripper design [68] (9) study of acoustic properties and behavior of LCNEs [71,72,73]
MC	(1) performing large-scale computer simulations for studying the effect of electric field as an actuation tool, to clarify the operational behavior of the LCEs on a molecular level [83] (2) developing MD method that could capture and reproduce experimental results obtained from a variety of experiments including stress strain, order, light transmission and thermal effects [85] (3) studying biaxial LCEs and predicting calorimetry data and deuterium magnetic resonance spectra [86] (4) investigating how sample preparation affects the nematic-isotropic behavior in LCE samples with both regular and irregular polymer networks [88] (5) gaining a better understanding of the anisotropic phenomena exhibited by LCEs [90] (6) Investigation of Shape and volume phase transitions of LCEs for both swollen and non-swollen state [97]
MD	(1) predict the way the mechanical response of LCEs with side-chain architectures depends on their chemical structure [60] (2) studying how phase transition from monodomain to polydomain occurs in LCEs on a molecular scale [8] (3) developed a multiscale framework for studying the photomechanical behavior of photo-responsive polymer networks [103] (4) developed a coarse-grained model consisting of mesogenic molecules and smeared charges for the study of Liquid crystal polymers (LCPs) [107] (5) studying which force field among the three, Deriding, PCFF, and SciPCFF potentials should be applied to LCE modeling system [110]
Other numerical methods	(1) exploring the dynamical behavior of LCEs [111,112] (2) model thermal–order–mechanical coupling behaviors in LCEs [36] (3) modeling the mechanical procedure by which biological cells exchange information [120] (4) studying the mechanical behavior of Smectic-A LCEs, in response to electromechanical stimuli [124] (5) modeling the electro-elastic behavior of nematic LCEs [128] (6) studying the thermos-mechanical behavior of a soft robot [133] (7) developing a general frame work for studying the interactions between LCs and polymer backbones that constitute a LCE [134]

**Table 2:** Categorization of the reviewed numerical methods.

Method	Methodology and Formulation
FEM (software)	(1) the authors used an equation proposed by [35] and subsequently derived the linear stress strain relation for soft LCEs[34] (2) By using a non-linear theory developed by Warner and Terentjev [38], in conjunction with stretch test data, the authors were able to derive a linear model for carrying out their numerical analysis on nematic elastomers [37] (3) A modified linear version of the Warner and Terentjev [38] model was used by adding a spatially random temperature fluctuation term [38]. (4) The authors’ developed an empirical model, describing material deformation, using experimental actuation data [41]. (5) user-defined subroutines were developed [25]. (6) a shell conducting model with moving heat source was used [43]. (7) a phase-field model, for continuum-mechanical modeling of nematic LCEs, was implemented into the software [24] (8) A Koiter-type theory was developed, containing two terms: 2D or plane stress alleviation of the relaxed energy of DeSimone and Dolzmann [49] and bending [45] (9) a model first used by [26] was implemented, with two modifications: (a) defining the order parameter and nematic directors in each mesh element (tetrahedral in this case) used for discretizing the sample geometry, (b) insuring that enough number of mesh elements were used so that the directors transitioned smoothly [52]. (10) a statistical definition of microstructure nature of liquid crystal elastomers was presented then a quantitative, physics-based micromechanical model was used [53]
FEM (in house codes)	(1) a coarse-grain model was presented which used the definition of energy density[56]. (2) Hamiltonian FEM approach was utilized [26]. (3) instead of a linear strain tensor, a rotationally invariant Green-Lagrange strain tensor was used for deriving the Hamiltonian [27] (4) Linear brick elements were used to perform a low fidelity FE analysis [60] (5) the moving asymptotes (MMA) method was utilized [61] (6) Hybrid particle FE elastodynamics simulations, with tetrahedral meshes, were used [62] (7) based on the VWT energy description, two separate contributing terms for the elastic energy were considered, namely deformation of the nematic director and the other, representing elastic component [69] (8) hyper-elastic model was solved using mixed FEM [68] (9) a transient model was developed that could couple elasticity and hydrodynamics of nematic elastomers [71]. A theory for approximating the behavior of acoustic waves in nematic elastomers was developed [72] two approaches were implemented and tested: one was according to the linear continuum theory of nematic rubber elasticity and in the second, a constitutive relation for rubber behavior was obtained using interpolation of experimental master curve of regular material [73]
MC	(1) A semisoft deformation Monte Carlo method, in which the applied external field was perpendicular to the nematic director was used. all liquid crystal molecules were represented by uniaxial ellipsoids and The total interaction energy was obtained by summing up all non-bonded and bonded intermolecular contributions, using Soft-Core Gay–Berne Interaction [83]. (2) a simple coarse-grained lattice model was proposed. By obtaining the total Hamiltonian for LCE and through conducting constant-force Monte Carlo simulations, the simulations were carried out [85]. (3) a coarse-grain model was developed by summing of pseudo-Hamiltonians describing rubber elasticity, anisotropic interactions between biaxial mesogenic units, and the strain-orientational coupling of the polymeric chains [86] (4) main-chain systems were utilized. soft-core GB potential was used for the simulations. Uniaxial soft-core GB ellipsoids were utilized for modeling mesogenic molecules and for assembling LCE networks, and also to illustrate non-bonded swelling monomers [88] (5) Finsler geometry was used for the simulations [90]. A detailed description of Finsler geometry can be found in [91,92]. (6) the Finsler geometry was expanded by adding an Ising-like variable to account for both swollen and nonswollen LCE states [97]
MD	(1) The elastomer is formed by crosslinking of the melt in the smectic A phase [60] (2) a coarse grain model was used in which ellipsoidal shapes were used to describe the mesogens particles. In addition, Gay-Berne (GB) potential was used to describe the interactions between mesogens [8] (3) The MD method used, is based on the work of Choi et al. [105] and utilizes energetic relaxation and multi-step crosslinking. In order to fully take into effect, the influence of kinked cis- molecules, Landau expansion was substituted for Maier-Saupe phase transition [6] by using a modified heuristic equation [106]. (4) the intermolecular interaction between ellipsoids is modelled using the SCGB potential. Subsequently, for the calculation of the piezoelectric tensor, point charges were introduced. The simulations were done using a course-grained MD program COGNAC [108,109]. (5) LAMMPS was used to find the most appropriate force field for LCE modeling [110]
Other numerical methods	(1) non-local continuum model was developed using a novel preconditioner based on Chebyshev spectral collocation method [111,112] (2) total energy was a function of deformation gradient, director direction, order parameter and biaxiality. After deriving equations for mechanical and phase equilibrium, total free energy was considered to be a hybrid of the entropy-induced elastic energy and the Landau– de-Gennes nematic energy [36] (3) soft matter cell model based on Lagrange-type mesh-free Galerkin formulation was developed [120] (4) A free energy density function was obtained by summing contributions from (a) elastic free energy, (b) change in the layer thickness (c) variation in chirality, or equivalently the tilt (or rotation) of the director, (d) energetic contribution of the electrical sources to the total energy and (e) external mechanical loading [124] (5) a continuum-mechanical phase-field approach was adopted. A nemato-electro-elastic model is coupled with the fundamental Landau-de-Gennes theory for isotropic–nematic phase transition [128] (6) transient analytical solution consisting of a one dimensional heat transfer equation (solved using Green’s function) coupled with a bilayer beam model [133] (7) A continuum mechanics model was developed that in addition to taking into account the director or order tensors, along with their respective time derivatives, the deformation gradient and its derivative with respect to time was also considered [134]
